# Arterial Remodelling in Chronic Kidney Disease: Impact of Uraemic Toxins and New Pharmacological Approaches

**DOI:** 10.3390/jcm10173803

**Published:** 2021-08-25

**Authors:** Nabil Foudi, Maeva Palayer, Marie Briet, Anne-Sophie Garnier

**Affiliations:** 1 INSERM, CNRS, MITOVASC, Equipe CarMe, SFR ICAT, Université Angers, F-49000 Angers, France; nabil@foudi.com (N.F.); m.palayer@hotmail.fr (M.P.); annesophie.garnier@chu-angers.fr (A.-S.G.); 2 Service de Pharmacologie-Toxicologie et Pharmacovigilance, Centre Hospitalo-Universitaire d’Angers, F-49000 Angers, France; 3 Service de Néphrologie-Dialyse-Transplantation, Centre Hospitalo-Universitaire d’Angers, F-49000 Angers, France

**Keywords:** chronic kidney disease, arterial remodelling, uraemic toxins

## Abstract

Chronic kidney disease (CKD) is a major public health concern that affects around 10 percent of the world’s population. The severity of CKD is mainly due to the high prevalence of cardiovascular (CV) complications in this population. The aim of this review is to describe the arterial remodelling associated with CKD, to provide a quick overview of the mechanisms involved and to review the recent pharmacological approaches aimed at improving vascular health in CKD. CKD patients are exposed to metabolic and haemodynamic disorders that may affect the CV system. Large artery functional and geometric abnormalities have been well documented in CKD patients and are associated with an increase in arterial stiffness and a maladaptive remodelling. Uraemic toxins, such as indoxyl sulphate, p-cresyl sulphate, protein carbamylation and advanced glycation products, exert various effects on vascular smooth muscle cell functions. The low-grade inflammation associated with CKD may also affect arterial wall composition and remodelling. It is worth noting that the CV risk for CKD patients remains high despite the pharmacological control of traditional CV risk factors, suggesting the need for innovative therapeutic strategies. An interventional study targeting the NLRP3 inflammasome has provided some interesting preliminary results that need to be confirmed, especially in terms of safety.

## 1. Chronic Kidney Disease, Definition and Epidemiology

Chronic kidney disease (CKD) is a major public health concern due to its prevalence and severity. Eleven to thirteen percent of the world’s population is affected by CKD [1], and this percentage is expected to grow in the next few years because of an increase in the prevalence of diabetes mellitus, hypertension and obesity [2]. Between 2010 and 2017, CKD increased from the 27th to the 17th most prevalent cause of death in the world according to the Global Burden of Disease study [3].

CKD is defined as abnormalities of kidney function or structure, present for at least 3 months, with implications for health [4]. In 2012, the Kidney Disease: Improving Global Outcomes (KDIGO) working group published a classification of CKD based on the following criteria: cause of CKD, glomerular filtration rate (GFR) and presence of albuminuria. The combination of these criteria can be used to stratify the risk of progression to end-stage renal disease (ESRD) and the risk of complications such as cardiovascular (CV) events, infections, hospitalisations and death [4].

Diabetes mellitus and hypertension are the leading causes of CKD in all developed and many developing countries [5]. Environmental pollution, pesticides, herbal medications and infections contribute to the occurrence of CKD in low-income countries. By contrast, inactivity, obesity and their consequences on health are the main factors in developed countries [6].

## 2. Cardiovascular Risk and Chronic Kidney Disease

Cardiovascular (CV) diseases are the main cause of death and morbidity in patients with CKD [7]. CV mortality is 10 to 30 times higher in ESRD compared with that in the general population [8], and this risk is even higher for young adults with ESRD. There is a graded inverse relationship between CV risk and GFR [9]. In addition, epidemiological studies have shown that patients with a preserved GFR and isolated microalbuminuria also have a higher risk of CV mortality than the general population [10]. The importance of CV complications in CKD patients is illustrated by the fact that CKD patients are more likely to die from CV diseases than to progress to ESRD. The risk of kidney failure may exceed the risk of CV events only for patients with a severely impaired renal function (stage 4) [11]. 

The leading causes of CKD, such as diabetes and hypertension, only partly explain the high level of CV risk in CKD patients. Indeed, meta-analyses have shown that a low GFR and increased albuminuria are both independent CV risk factors regardless of the underlying disease [12,13].

Furthermore, the epidemiology of CV diseases in CKD patients is different than in patients with a high CV risk but with normal kidney function. For instance, heart failure is three to four times more common in CKD patients than in patients with preserved GFR [14]. Around 55% of haemodialysis patients have heart failure [15]. The risk for CKD patients is also higher in cases of stroke, peripheral artery disease, coronary heart disease and atrial fibrillation [11].

The mechanisms linking CKD and CV are several and complex. An alteration in kidney structure and/or function leads to haemodynamic and metabolic disorders that can affect the CV system. These uraemia-related CV risk factors include, among others, oxidative stress, inflammation, insulin resistance and accumulation of toxins that are usually excreted by the kidneys. As a consequence, preventing CV diseases through interventions that focus only on traditional risk factors may not be sufficiently effective in preventing CV diseases. Indeed, in patients with ESRD, interventional studies focussed on traditional CV risk factors such as dyslipidaemia failed to improve survival rates [16,17,18,19,20].

## 3. Arterial Remodelling Associated with CKD

Large artery functional and remodelling abnormalities have been well documented in patients with ESRD. In 1990, London et al. showed that aortic stiffness, determined by the measurement of aortic pulse wave velocity (PWV), and aortic diameter were significantly increased in ESRD patients [21]. In this population, arterial stiffness is a strong independent predictor of all-cause and CV mortality [22]. In addition, the improvement of arterial stiffness over time in ESRD has been associated with a better prognosis. Longitudinal follow-up of ESRD patients showed that arterial stiffening progresses rapidly over time in haemodialysis patients. In a Canadian prospective cohort, the annual rate of change in carotid–femoral PWV was 0.84 m/s per year [23]. In fact, arterial remodelling also occurs from the earliest stages. Several clinical studies have shown a graded association of arterial stiffness with GFR in patients with more advanced CKD from stages 2 to 5, compared to that in hypertensive patients or healthy subjects [24,25]. Arterial stiffness is also an independent risk factor for mortality, heart failure and progression in patients not yet dialysed [26,27,28]. In addition to increased arterial stiffness, CKD at stage 2–5 is associated with an increase in arterial diameter and circumferential wall stress. In a longitudinal follow-up study involving CKD patients at stages 3–5 with a mean follow-up of 3.1 years, these two parameters progressed over time [29] and were associated with the progression towards ESRD, all-cause mortality and CV prognosis [30]. 

## 4. Impact of Uraemic Toxins on Arterial Remodelling

**Figure 1:**
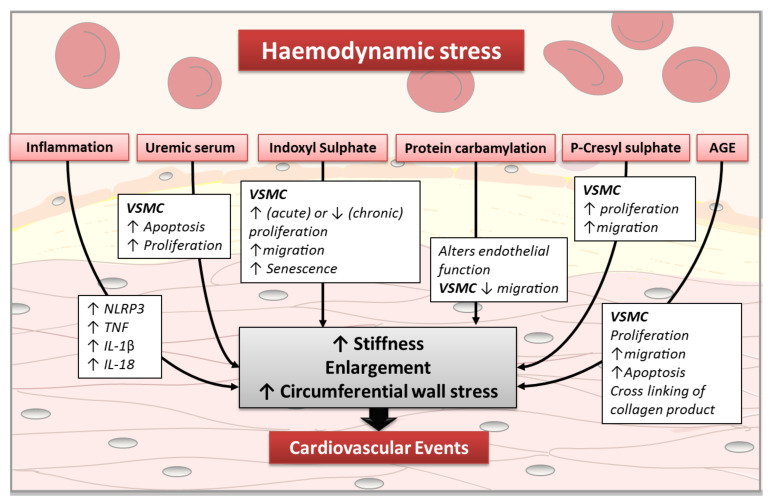
Impact of uraemic environment on arterial remodelling. VSMC: vascular smooth muscle cell;AGE: advanced glycation product.

In cases of CKD, the risk of CV mortality is not fully explained by the presence of traditional CV risk factors and probably has a multifactorial origin. Among the factors involved, the presence of uraemic toxins may affect the arterial structure and function and contribute to the occurrence of CV diseases (Figure 1). The impact of mineral and bone metabolism disorders on vascular smooth muscle cells (VSMCs) and arterial remodelling has been extensively described in many reviews [31,32,33,34] and is not detailed here.

## 5. Uraemic Toxin Definition and Classification

The uraemic syndrome is characterised by the retention of various solutes that would normally be excreted by the kidneys. Among them, substances that interact negatively with biologic functions are called uraemic toxins. Since 1999, the European Uraemic Toxin (EUTox) working group has been looking at the identification and classification of uraemic toxins. The most widely used classification of these toxins is based on their molecular weight and on the pattern of removal by means of dialysis, one of the strategies that remains the most effective in reducing their concentration [35]. This classification is made up of three groups. The first group are the small, water-soluble uraemic toxins. This group includes small molecules with a molecular weight below 500 Da, which are soluble in water and easily removed by means of dialysis. These small toxins have been associated with CV diseases and inflammation [36]. Among them, uric acid, asymmetric dimethylarginine and urea and its metabolites have been associated with all-cause mortality and the occurrence of CV events in CKD [37,38,39]. The second group are the medium-sized molecules with a molecular mass greater than or equal to 500 Da. Due to their mainly endogenous origin, their concentration is not only increased by their retention but also by endocrine and paracrine homeostasis mechanisms. Their extra-renal purification, therefore, requires adapted equipment. Their impact has been primarily described in CV diseases, inflammation and fibrosis [36]. Among these molecules, we can mention β-2 microglobulin, the most documented molecule, which is involved in many physiopathological phenomena including arterial stiffness in cases of CKD [40], and endothelin, which is associated with increased blood pressure and may be associated with renal inflammation, oxidative stress, vascular shear stress, arterial stiffness, endothelial dysfunction and atherosclerosis [41,42]. Finally, the third group is made up of the protein-bound solutes. This is a heterogeneous group composed of small molecules mainly of intestinal origin [36] that are recognised as being difficult to remove by means of dialysis because of their protein-binding capacity. The best-documented substances are the advanced glycation end products (AGEs) and the group of kynurenines. Both are associated with the occurrence of CV events in CKD patients [43,44]. 

## 6. Impact of Uraemic Toxins on Vascular Smooth Muscle Cell Functions

Uraemic toxins may induce vascular damage directly or indirectly, through various mechanisms involving, among others, the production of reactive derived products or the post-translational modification of proteins, such as glycation or carbamylation. Here, we briefly review our current knowledge about their impact on VSMC functions (Table 1).

### 6.1. Effect of Uraemic Serum on VSMC Functions

A few studies have evaluated the effect of uraemic serum on VSMC functions, with contradictory results. Some authors have demonstrated that human aortic VSMCs exposed to uraemic serum exhibited decreased viability, increased apoptosis and proliferation [48], whereas in another work, exposition of VSMCs to uraemic conditions had no effect on apoptosis and induced a decrease in proliferation [45]. A decrease in VSMC migration was observed in two published studies in the context of uraemic serum exposure [45,48]. The impact of uraemic serum on senescence needs to be clarified (Table 1).

### 6.2. Effect of Indoxyl Sulphate (IS) 

The effect of indoxyl sulphate on VSMCs depends on the duration of exposure. Acute exposure (1 day) to IS has been associated with an increase in VSMC proliferation [51,52], whereas chronic exposure (7 days) induced a significant inhibitory effect on human VSMC proliferation [53]. In addition, acute exposure to IS has also been associated with an increase in VSMC migration [55] and promoted VSMC senescence with an upregulation of p53 and p21 in an oxidative-stress-dependent mechanism [58]. In addition, a recent experimental study demonstrated that IS promoted the calcification of human aortic smooth muscle cells through PI3K/Akt/NFκB signalling [59]. 

### 6.3. Effect of P-Cresyl Sulphate (PCS) 

The impact of PCS on VSMC function has been little described in literature. In vitro, PCS exposure did not alter VSMC proliferation [47]. However, in vivo, PCS has been shown to facilitate the migration and proliferation of VSMC in atherosclerotic lesions in ApoE^−/−^ mice that underwent 5/6 nephrectomy [54]. Thus, more studies are required to evaluate the effects of PCS not only on VSMC proliferation but also on apoptosis, migration and senescence.

### 6.4. Impact of Protein Carbamylation on Vascular Function

Accumulation of isocyanic acid—a reactive derived product of urea—in the context of CKD can lead to protein carbamylation, a post-translational modification characterised by the spontaneous binding of isocyanic acid to free amino groups of proteins or amino acids [60]. Protein carbamylation is a significant and independent predictor of mortality and CV events in ESRD patients [39]. In an experimental model of ApoE^−^/^−^ mice subjected to unilateral nephrectomy and a high-fat diet, carbamylated low-density lipoproteins (LDLs) have been associated with the development of atherosclerosis [61]. In another experimental study, exposure to cyanate by inhalation led to aortic endothelial dysfunction in vivo. In vitro, carbamylation of human umbilical vein endothelial cells (HUVECs) has been associated with a decrease in the expression of endothelial nitric oxide synthase [62]. In addition, the effect of carbamylation of intracellular proteins has been recently described [63]. The accumulation of intracellular carbamylated proteins in fibroblasts exposed to sodium cyanate did not affect cell proliferation or senescence but significantly decreased cell motility [63]. Protein carbamylation may also affect arterial remodelling, but to date, its impact on VSMC functions has not been described in literature. 

### 6.5. Effect of Advanced Glycation End Products (AGEs) on VSMC Functions

Glycation is defined as the binding of sugar carbonyl groups to free amino groups of proteins, leading to the generation of AGEs after exposure to irreversible oxidative reactions [60]. Several studies have demonstrated a significant association between AGE and arterial stiffness in ESRD patients [64,65,66]. AGEs have also been involved in uraemic vasculopathy. AGEs exhibited biphasic effects on the proliferation of rabbit VSMCs, increasing or decreasing proliferation depending on the dose used during exposure [67]. Moreover, AGEs seem to play an important role in VSMC migration and apoptosis. In vitro, the activation of the AGE receptor (RAGE) by AGEs induced VSMC migration [68]. AGEs have been shown to stimulate VSMC apoptosis through excessive reactive oxygen species (ROS) generation [69]. In addition to the effect on VSMC proliferation, migration and apoptosis, AGE also induces crosslinking of collagen products, which may affect the arterial phenotype [70].

## 7. Inflammation and Arterial Remodelling in the Context of CKD

CKD is considered to be a pro-inflammatory condition. An increase in inflammatory markers has been regularly reported in CKD patients, particularly at advanced stages [71,72]. Increasing evidence suggests that inflammation plays an important role in the progression of vascular remodelling leading to a wide range of CV diseases. Indeed, inflammatory reactions are among the primary events that are thought to modulate cell processes involved in vascular remodelling such as extracellular matrix synthesis and degradation, apoptosis, senescence, proliferation and migration of VSMCs, leading to a disturbance in the structural and functional integrity of the vascular wall. Inflammatory mediators such as the tumour necrosis factor (TNF) affect NO synthase function leading to the production of reactive oxygen species [73]. This is accompanied by a phenotypic switching of VSMCs, the production of matrix metalloproteinases, the inhibition of the tissue inhibitors of matrix metalloproteinases and extra-cellular matrix alterations [74].

In CKD patients, increased levels of IL-6 and TNFα levels have been associated with aortic stiffness [75,76,77]. In another clinical study, a significant positive correlation has been observed between the expansion of CD14^+^CD16^+^ monocytes, the high-sensitive C-reactive protein levels and the brachial-ankle pulse wave velocity [72]. An increase in C-X-C Motif Chemokine Ligand 12 (CXCL12) has been shown in CKD patients and associated with left ventricular hypertrophy, hypertension, and CV events such as myocardial infarction [78,79]. CC-chemokines are synthetised by leucocytes and work through the activation of CC-chemokine receptors (CCRs). The expression of CCRs by vascular cells suggests there is a direct effect of CXCL12 on these cells [80]. 

The low-grade inflammation observed in CKD patients may be related to the NOD-like receptor family pyrin, which leads to domain-3 (NLRP3) inflammasome activation. The NLRP3 inflammasome is a complex associating the intra-cellular sensor NLRP3, the adaptor apoptosis-associated speck-like protein (ASC) and procaspase-1. The assembly of inflammasome leads to the cleavage of procaspase-1 into active caspase-1 that proteolytically cleaves the cytokine precursor prointerleukin-1β (pro-IL-1β) and prointerleukin-18 (pro-IL-18) into biologically active IL-1β and IL-18 [81]. In CKD patients, an increase in IL-1β in plasma and in PBMC has been reported [82,83]. Experimental models, particularly hypertension models, suggest that NLRP3 inflammasome activation contributes to changes in vascular composition and remodelling [84,85]. These observations suggest a potentially central role of inflammation and the NLRP3 inflammasome pathway in arterial remodelling.

## 8. Pharmacological Approaches of Uraemia-Linked Arterial Remodelling

Only a few trials have been conducted in the CKD population evaluating whether the management of CV factors efficiently decreased the CV risk in CKD patients. Evidence of lipid-lowering treatment has been demonstrated in non-dialysis CKD patients [86] but not in ESRD patients [16,18]. It is worth noting that the CV risk of CKD patients remains high despite the pharmacological control of traditional CV risk factors [87]. Recent or ongoing interventional trials conducted in these high-CV-risk patients evaluate new pharmacological approaches as described below.

As specified earlier, chronic inflammation seems to play a significant role in the progression of vascular remodelling. Several highly active pro-inflammatory cytokines, such as IL-1 or IL-6, are considered to be early triggers of the inflammatory response and represent an interesting target in improving the CV risk associated with CKD [88]. In a two-site, double-blind trial, 42 patients with stage 3–4 CKD were randomised to receive rilonacept, an IL-1 trap, or placebo for 12 weeks. Primary end points were changed in brachial artery flow–mediated dilation (FMDBA) and aortic pulse-wave velocity (aPWV) after 4, 8 and 12 weeks. Overall, 24% of patients were women, the mean age was 63 ± 11 years and the mean eGFR was 38 ± 13 mL/min per 1.73 m^2^. Compared to placebo, rilonacept significantly improved FMDBA (*p* < 0.01), without changing aPWV (*p* = 0.56). Rilonacept also reduced hsCRP levels (*p* < 0.01) and endothelial cell NADPH oxidase expression (*p* < 0.05). Five adverse events were observed in the rilonacept group versus two with placebo [88].

Anakinra^®^, a recombinant human IL-1 receptor antagonist, has also been shown to reduce systemic inflammation in ESRD patients, inducing a significant reduction of human serum C-reactive protein (hsCRP) (*p* = 0.008) and IL-6 levels (*p* = 0.03) compared to placebo [86]. The safety and tolerability of anakinra^®^ in CKD patients will be evaluated in the ACTION trial, a phase II multicentre study with 80 ESRD patients on maintenance haemodialysis. Participants are randomised to receive either anakinra^®^ 100 mg administered intravenously 3 times per week at the end of the haemodialysis session or placebo for 24 weeks. In addition to the side effects, other outcomes will be evaluated, including circulating C-reactive protein (CRP) concentration, circulating markers of inflammation, oxidative stress and cardiac disease and nutritional and metabolic markers [89].

Finally, Van Tassell et al. designed a phase 2 interventional single-arm trial in order to evaluate the effect of Anakinra^®^ on cardiorespiratory fitness in patients with advanced chronic heart failure (left ventricular ejection fraction less than 50%) and stage 4–5 CKD. The outcomes were peak oxygen consumption during cardiopulmonary exercise and hospitalisation due to heart failure during the 6 months of treatment. Unfortunately, no results are available because the study was withdrawn due to the lack of participants.

Various other strategies aimed at improving the vascular health of CKD patients are currently under evaluation including drug repositioning—clopidogrel, hydroxychloroquine, metformin—or new molecules such as KBP-5074. These trials are summarised in Table 2.

In conclusion, CKD is associated with multiple metabolic and haemodynamic abnormalities that may affect arterial structure and functions. The prognosis value of arterial stiffness and remodelling parameters suggest that their evaluation could be of interest in this population. Traditional therapeutic approaches only improve the prognosis of these patients partially, reinforcing the need to identify new pharmacological targets.

## Figures and Tables

**Table 1:** Impact of uraemic toxins on VSMC functions.

	Uraemic Serum	Phosphate	Indoxyl Sulphate Acute Exposure Chronic Exposure		P-Cresyl Sulphate
**Viability** Primary HASMC	Decrease [45]	Decrease [46]			No effect [47]
**Proliferation** Primary HASMC Others	Slight decrease [45] or increase [48] Increase/aortic explants [49]	Decrease [46,50]	Increase [51,52]	Decrease [53]	Increase [54]/rat aortic VSMC
**Apoptosis** Primary HASMC	Increase [45] or no effect [48]	Increase [46]	Not evaluated		Not evaluated
**Migration** Primary HASMC Others	Decrease [45,48] Increase [49]/aortic explants	Acceleration [50]	Increase [55]		Increase [54]
**Senescence** Primary HASMC	Needs to be clarified	Increase [46,56,57]/cell cycle arrest	Promotion [58]		Not evaluated

**Table 2:** Currently recruiting clinical trials evaluating drug effects on vascular remodelling during chronic kidney disease (data from clinicaltrials.gov). CKD: chronic kidney disease, NA: not applicable, NCT: national clinical trial.

Study Start	Study Title	Population Studied	Drugs and Procedures	Phase	Locations	NCT #
March 2020	Oral Absorbent and Probiotics in CKD Patients With PAD on Gut Microbiota, IncRNA, Metabolome, and Vascular Function	CKD all stages	Dietary supplement	NA	Taiwan	04792320
November 2019	Nicotinamide Riboside Supplementation for Treating Arterial Stiffness and Elevated Systolic Blood Pressure in Patients with Moderate to Severe CKD	CKD stage 3 to 4	Drug: nicotinamide riboside Primary outcome: change in aortic stiffness (carotid–femoral PWV) Follow-up: 3 months	Phase 2	Denver, CO, USA	04040959
December 2018	HCQ for the CVD in CKD	CKD stage 3B, 4	Drug: hydroxychloroquine Outcomes: - carotid total plaque volume (carotid MRI) - aortic stiffness (aortic PWV) Follow-up: 18 months	Phase 2	Gainesville, FL, USA	03636152
April 2018	Phase 2b Study of KBP-5074 in Subjects with Uncontrolled Hypertension and Advanced Chronic Kidney Disease	CKD stage 3B, 4, uncontrolled hypertension	Drug: KBP-5074 (mineralocorticoid receptor antagonist) Outcome: systolic blood pressure Follow-up: 84 days	Phase 2	Princeton, NJ, USA Morrisville, NC, USA	03574363
September 2017	Vitamin K to Slow Progression of Cardiovascular Disease Risk in Haemodialysis Patients	Stages 3, 4 and 5 of CKD	Drug: menaquinone-7 Outcomes: - endothelial function (flow-mediated dilation) - aortic stiffness (AoPWV) Follow-up: 8 weeks	NA	Augusta, GA, USA	03311321
July 2017	Effectiveness and Tolerability of Long-Acting Nifedipine Gastrointestinal Therapeutic System in Chronic Kidney Disease with Uncontrolled Hypertension Patients, a Prospective, Multicentre, Observational Study	CKD, uncontrolled hypertension	Drug: nifedipine controlled-release tablets (Adalat, BAYA1040) Outcome: systolic blood pressure Follow-up: 12 weeks	NA	Multiple locations, China	03194633
April 2017	Study Comparing Treatment Effectiveness of Guideline Indicated APT for ACS in Patients with CKD	CKD, acute coronary syndrome	Drug: ticagrelor Drug: clopidogrel Outcome: occurrence of all-cause mortality, non-fatal myocardial infarction, stroke	Phase 4	Durham, NC, USA Dallas, TX, USA	03150667
October 2014	Metformin in Kidney Disease	CKD stage 3 overweight or obese, normal if pre-diabetic or insulin resistant	Drug: metformin Outcome: leptin to adiponectin ratio (an atherosclerotic index) Follow-up: 16 weeks	Phase 2	Nashville, TN, USA	02252081
